# Impact of Entrepreneurship on the Quality of Public Health Sector Institutions and Policies

**DOI:** 10.3390/ijerph19031569

**Published:** 2022-01-29

**Authors:** Jelica Rastoka, Saša Petković, Dragana Radicic

**Affiliations:** 1 Deposit Insurance Agency of Bosnia and Herzegovina, 78000 Banja Luka, Bosnia and Herzegovina; jelica_rastoka@aod.ba; 2 University of Banja Luka, Faculty of Economics, 78000 Banja Luka, Bosnia and Herzegovina; sasa.petkovic@ef.unibl.org; 3 Lincoln International Business School, Lincoln LN6 7TS, UK

**Keywords:** public health entrepreneurship, entrepreneurial orientation (EO), EO dimensions, public healthcare infrastructure, health service efficiency

## Abstract

The purpose of this paper is to investigate whether public health entrepreneurship principles implementation in the public health sector are alternative ways of promoting an immediate improvement of healthcare infrastructure. To contribute to the literature on the impact of public health entrepreneurship on public healthcare infrastructure, we estimate two empirical models, with the first model having institutions and the second model having public healthcare policies as the dependent variable. Our empirical analysis is based on the WHO international health regulation data for all WHO member countries (in order to achieve a balanced panel, we decided to retain 192 of them), covering the period from 2010 through to 2019. The main results obtained using a Poisson panel regression indicate a positive relationship between employing more entrepreneurship within public healthcare and the quality of public healthcare infrastructure represented through institutions and policies. This study produces several contributions to the stream of research on public health entrepreneurship. First, it makes a theoretical contribution in the way that it fills the lacking literature on the relationship between entrepreneurship within the public health sector and efficiency of country-specific public healthcare infrastructure. Second, it offers an empirical quantitative analysis of entrepreneurship that is generally lacking. Concerning policy implications, the third contribution of this paper is the provision of evidence showing alternative ways to improve healthcare infrastructure other than traditionally observed investments in physical infrastructure.

## 1. Introduction

In 2020, the outbreak of coronavirus threatened to demolish public healthcare systems all around the world. As reported “the global and rapidly spreading virus has placed unprecedented demands on health systems in most countries around the world” [1] (p. 1). With public healthcare being on the verge of a collapse, the question of whether and how is it possible to improve healthcare infrastructure naturally arose. The first answer that comes to mind would be investing more funds, extending physical and human capacities, etc. However, a number of limitations that countries faced (especially in terms of time and budget) made this issue more puzzling than ever. As it later turned out, the size of physical infrastructure was not fully commensurate with the success a country had with handling the crisis. Today, almost 2 years from the beginning of the global pandemic, even though it seems that the worst is behind us, apparently a number of other threats are lurking—in the shape of new strains and variants of coronavirus, or perhaps even other viruses. Either way, it is very likely that public healthcare systems are yet to face pressures in terms of rising demands for better provision and higher quality of healthcare services. In general, the literature on these topics contends that one of the main determinants of a healthcare quality is the level of development of a health infrastructure, which is globally deemed to be underdeveloped [2,3]. These issues are particularly pronounced in less-developed countries facing more constraints in terms of capacities for improving healthcare systems by simply injecting money. Moreover, bearing in mind a generally lower level of their population’s health, less-developed countries are naturally more susceptible to health crises which may induce a cascade of further socio-economic issues through negative spill-over effects. Thus, motivated by the current situation, the question we seek to answer in this study is as follows: *Does implementation of entrepreneurship principles influence the enhancement of public health infrastructure?*

In our attempt to answer this question, we follow the model of Hall and Jones [4], who first introduced social infrastructure through institutions and policies. Analogous to that, we decompose public healthcare infrastructure via public healthcare institutions and policies, thereby focusing strictly on intangible infrastructure (that is, institutions in terms of norms and rules, while policies are regarded as a course or a principle of action). With *enhancement* in public health infrastructure, what we have in mind is an increase in their quality. Now, the quality of the public healthcare sector is somewhat more ambiguous compared to the other industries. Namely, as public healthcare is both a government sector as well as non-profit, the quality of this industry shall not be assessed using mainly accounting measurements. Thus, the quality of the public healthcare sector shall be observed through their provision of services and overall performance. As the ultimate goal of any public healthcare system is preventing health risks, promptly responding to emergencies, and generally providing better health to a nation, hence the *enhancement* in its infrastructure could be understood as an improvement in the quality of their provision of health services, fighting health risks, and answering to health emergencies. Finally, we ask ourselves what might influence the quality of public healthcare’s intangible infrastructure (hereinafter: public healthcare infrastructure, or PHI). From the immense literature on entrepreneurship [5,6,7,8,9,10], we accept cogent arguments of entrepreneurship positively affecting the organization implementing it per se, as well as the outcomes it produces.

Putting these results together suffices to suspect the positive correlation between entrepreneurship and public healthcare infrastructure, though we found no literature describing this particular relationship. That is, we found no theoretical nor empirical evidence studying the relationship between fostering public health entrepreneurship [11] within public healthcare on the one side, and the quality of public healthcare infrastructure in terms of institutions and policies [12,13] on the other side.

To contribute to the literature on the impact of public health entrepreneurship on public healthcare infrastructure, we estimated two empirical models, with the first model having institutions and the second model having public healthcare policies as the dependent variable. For each of our two models, we estimated four different specifications, taking into account the relevant literature on entrepreneurship. More specifically, we decided to estimate each model under the following two approaches: (i) the single-dimension approach to studying entrepreneurship (arguing entrepreneurial values covariate, moving roughly around the same level) [14,15,16], and (ii) the multidimensional approach (arguing entrepreneurial variables move freely from each other, allowing for the difference between their levels to be significant) [17,18]. Furthermore, we decided to tackle the ever-going discussion on how many dimensions shall be included in studying entrepreneurship, taking into account two general points of view: (i) the first, arguing that three main dimensions—innovation, proactivity, and risk taking—capture most of entrepreneurship, so adding more dimensions is redundant; (ii) the second viewpoint, agreeing with the first in part that adding too many entrepreneurial variables produces noise, but deeming autonomy and competitive aggressiveness to be just as important as innovation, proactivity, and risk taking. Thus, for each of our two models, we estimated four model specifications.

Our empirical analysis is based on the WHO international health regulation data for all WHO member countries (in order to achieve a balanced panel, we decide to retain 192 of them), covering the period from 2010 through to 2019. The main results obtained using Poisson panel regression indicate a positive and statistically significant relationship between employing more entrepreneurship within public healthcare, and the quality of public healthcare infrastructure is represented through institutions and policies. 

This study produces several contributions to the stream of research on public health entrepreneurship. First, it makes a theoretical contribution in the way that it fills the lacking literature on the relationship between entrepreneurship within the public health sector and efficiency of country-specific public healthcare infrastructure. Second, it offers an empirical quantitative analysis of entrepreneurship that is generally lacking. We believe this to be a first study using WHO indices as proxies for public health entrepreneurship. Furthermore, it supplements the theory on approaches to measuring entrepreneurship (single vs. multidimensional approach) by offering a quantitative empirical analysis. Though abundant with theory, the literature discussing entrepreneurial variables (three dimensions vs. five or more dimensions) generally lacks empirical findings that would support theoretical frameworks. In terms of policy implications, the third contribution of this paper is evidence showing alternative ways to improve healthcare infrastructure other than traditionally observed investments in physical infrastructure. This is particularly important in the light of health crises urging for a prompt policy response. Namely, if entrepreneurship positively affects public healthcare infrastructure, policies would imply that triggering entrepreneurial acting within the public healthcare sector might improve its social infrastructure in the short run and, at least partially, amortize shocks. 

The remainder of this paper is structured as follows. The next section explains the theoretical framework together with the relevant empirical studies on entrepreneurship within the healthcare sector, studies of public health entrepreneurship within sectors different than healthcare, as well as studies of public entrepreneurship in general. The paper then follows with a description of the sample and data, as well as of the empirical strategy. Next, we present the main findings of our study, and discussions of main results. Finally, the paper concludes by outlining policy implications and offering directions for future research.

## 2. Literature Review

Global population ageing together with the outbreak of new technologies increase the expectations and possibilities of people in terms of healthcare provision [19]. The ever-growing demand for an increased level and diversification of healthcare services ignited interest in this topic not only among academia, but also international organizations which particularly stress the importance of healthcare, pointing out that a strong public healthcare sector is a key to increasing the quality of human capital [20]. A strong healthcare system requires a robust healthcare infrastructure [21,22]. As public healthcare has been recognized as an important element of the overall national healthcare system [23,24], it comes at no surprise that international organizations keep urging governments to increase investment in public healthcare and its infrastructure [25,26]. However, what is desirable might very likely be unfeasible for less-developed countries with budget constraints limiting their investment possibilities. Hence, we are interested in seeking alternative approaches for developing public healthcare and, thus, provision of a better healthcare service. We identify entrepreneurial behavior within public healthcare as a plausible driver of public healthcare infrastructure. Such intuition is underpinned with literature recognizing entrepreneurship as a genuine production factor, together with land, capital, and labour [27], as well as the abundant literature on the positive effects of entrepreneurship on public sector performance [28,29], introduction of new and transformation of existing institutions, processes, and products [30,31,32], improvement of quality of life, services, and value creation [33,34,35], but also enabling individuals and firms to become more involved in seizing opportunities, improving the environment [36,37,38], etc.

The second half of the 20th century spawned a number of papers on public entrepreneurship [39,40,41], and ever since, the interest on this topic has not waned [42,43,44,45], with the interest in healthcare entrepreneurship specifically also being maintained [7,46,47,48,49,50]. In their studies, scholars describe healthcare entrepreneurship as a specific mixture of innovativeness and healthcare diplomacy, as well as an important path to improving health, economic growth, and inclusive and sustainable development [51,52]. As they assert, healthcare entrepreneurship supports implementing entrepreneurial skills aimed at improving the public healthcare mission and broadening traditional approaches [29,53,54].

Despite the abundant literature on public entrepreneurship, as well as rapidly growing literature on healthcare entrepreneurship, rather less attention has been paid to these two topics combined; that is, public entrepreneurship within public healthcare systems. Most likely, one of the reasons for this stems from the overall lack of metrics for determining the level and scope of public entrepreneurship. Traditionally, internal entrepreneurship was deemed paradoxical, as entrepreneurship and corporate bureaucracy were considered mutually exclusive [55]. However, research that tested entrepreneurial values within existing business organizations suggest the opposite [56,57,58]. In this study, we adopt findings made by Heinonen [59] and regard entrepreneurship as an individual competency, focused on achieving certain goals, or a business orientation.

We underpin our theoretical arguments by the model of Hall and Jones [4], who first introduced the idea of social infrastructure being represented through institutions and policies; we choose to focus strictly on public healthcare institutions and policies. In this way, we postulate that *engaging in public health entrepreneurship increases the quality of public healthcare.*

One of the challenges with such studies is that sometimes the elements of public healthcare are not easy to determine, observe, measure, track, or quantify, and even if they were, the ethics of doing so would be questionable [60]. Even when there is some data on these elements, the problem very often lies in databases where these are stored, as they are usually—to a certain extent—incomplete, with quite limited access. Thus, it is generally deemed that there are no instruments for measuring healthcare outcomes that are easy to use [2]. In order to deal with this, we start by specifically describing what each public healthcare institution and policy represents and seeing if we can match it to the only global database owning data on public healthcare, the WHO database. 

With public healthcare institutions, we have in mind the formal and informal rules, norms, and procedures organizing the work of a public healthcare organization. That is, the institution in this context suggests a custom or tradition intrinsic to a certain national healthcare system (NHS). It does not suggest an organization, but a set of adopted practices. More specifically, with public healthcare institutions we have in mind a set of plans or a course of action an NHS develops in order to guide their actions, that is, provision of healthcare services. As explained in other studies [22], public health institutions provide a robust response system, combine trained staff, provide necessary laboratory capacity, and establish mechanisms for interaction, with all this being in line with international health regulations. Health policy is a set of activities directing and guiding healthcare activities and at the same time combining the available assets in such a way that would help meet all health-related and other national goals. Importance of policies arises from the intrinsic characteristics of public healthcare services. Namely, as with any other public good or service, while consuming it, one might prevent another individual from doing the same. As Ostrom [61] suggests, there are two possible solutions for this conflict of interest, which are either temporal or a physical separation. As decisions on optimal allocations are a matter of policies, this supports the views on healthcare quality being conditional upon health policies. Healthcare policies also help regulate healthcare systems [62]. In general, both institutions and policies in this context refer to intangible aspects of a public healthcare system of a country.

Our second challenge is defining the entrepreneurship within public healthcare and finding a way to appropriately quantify it. Though the research on public entrepreneurship evolved, most of it [34,62,63,64,65,66], as the authors themselves explain, study entrepreneurship only theoretically, whereas empirical research is still lacking. Likewise, even when research involves empirical evidence [56], it focuses on different organizational characteristics, most of which are not strictly connected with entrepreneurship but rather with a successful business in general, such as for example, customer relationships, work and reward system, strategic actions, learning, etc. Furthermore, a caveat with most studies on healthcare entrepreneurship [28,29] is not distinguishing between managerial and entrepreneurial practices, thus ending up with identifying managerial and organizational variables as “factors of entrepreneurship”. What these studies do is use qualitative reasoning to argue how managerial and organizational factors could be translated (i.e., equalized) into entrepreneurial factors. Another feature of these studies is strictly focusing on healthcare entrepreneurship within a single organization. If we look at studies of health infrastructure (regardless of entrepreneurship), we find that common measures of its quality are actually proxies, such as government spending, i.e., fiscal expenditure on healthcare [67]. However, as we are interested in non-tangible infrastructure, such measures would not serve as good proxies. 

In general, an entrepreneurial environment is believed to attract more entrepreneurship [68], hence many authors tend to choose the environmental elements for assessing entrepreneurship [69]. Most commonly, they use entrepreneurial culture [70,71,72], formal institutions [73], and informal institutions such as norms and social values [74,75]. Others, however, regard entrepreneurship as mostly predetermined by the individual capacity of an entrepreneur [76,77,78]. Such studies measure various indicators, such as socio-demographic, professional characteristics and motivational factors [79,80], personal competencies [46], psychological characteristics and cognitive abilities [81,82], etc. Still, an important thing to keep in mind is that in these cases, we predominantly consider private, market-oriented organizations. Finally, there are also opinions that entrepreneurship depends on the environment and characteristics of an entrepreneur, that is, a combination of these two [83].

A third approach to measuring entrepreneurship is performance-based, taking into account results and performance such as innovation and growth [76,84,85]. Recommendations for measuring outcomes of entrepreneurship in public institutions are either introducing or repealing regulations, resource reallocation, or similar actions [86]. For performance-based measurement, a general recommendation for choosing among different outcomes of entrepreneurship is opting for ones best representing overall implementation of entrepreneurial concepts of autonomy, innovation, proactivity, risk appetite, and competitive aggressiveness [66,87,88,89]. Papers on healthcare entrepreneurship that employed a performance-based approach usually measured innovation [90], as it can be much more easily quantified compared to other principles of entrepreneurship. A challenge with explaining and measuring the scope of public healthcare entrepreneurship arises from the nature of this category of entrepreneurship [29]. Hence, significant outcomes to be measured should refer to value added, innovation support, supporting healthcare in delivering its mandate [91], but also internal organizational characteristics such as management support, autonomy, awards, time availability, and organizational procedures [58,92].

Regardless of measuring approaches, conceptualization and operationalization of entrepreneurship is being performed through entrepreneurial orientation [93]. Most authors [14,62,93,94,95,96,97] use the so-called Miller‘s approach [89,98] for analyzing entrepreneurial orientation. According to this approach, the three main constructs of entrepreneurial orientation are as follows: innovativeness (in a sense of predisposition of developing new and unique products, services, and processes); risk-taking (readiness for seizing risky opportunities); and proactivity (persistence and creativity for overcoming difficulties while innovation being implemented fully). A further two dimensions are autonomy and competitive aggressiveness [87,99]. Although several authors also mention new ventures [100,101,102,103], new business activities, and self-improvement [104,105,106], an important thing to bear in mind is that adding too many dimensions of entrepreneurship might lead to collinearity issues, as certain dimensions overlap or are even in causal relationships. In order to decide on the number and which exact dimensions to use, we start from defining the five dimensions and finding support for using it.

A commonly mentioned feature of entrepreneurship is developing competitive advantage based on innovativeness [56,107]. Apart from competitive advantage, innovation enables the growth and development of an organization, while increasing employees’ satisfaction [108]. Furthermore, it enables institutional changes, so that an institution would not lose its relevancy and responsibility [33]. In public healthcare, innovation is usually perceived as a key strategy for stimulating transformations aimed at responding to a constantly growing demand for healthcare services, within limited finances [91]. Innovation in the health industry can be targeting either citizens or outcomes, depending on whether their goal is to increase overall health or they are targeting healthcare services and specific health institutions [109]. Lately, there has been much talk about promoting responsible innovation in health—RIH—that besides health goals, also takes care of other sustainable development goals and connects different stakeholders with the intention to achieve as large as possible positive effects for the whole community [110,111]. Due to observational limitations, innovation is usually measured through a product or service (procedures or techniques) innovation [88,103,106,112].

Proactivity is a manner of responding to opportunities from the environment [66,113,114], with anticipation of future problems, issues, or potential changes [112]. Most commonly it is seen as something “pioneering” [6] and taking initiative [87,88,105,114,115]. Proactivity is a key for developing new products and services, technologies and administrative techniques, and innovation in general [66]. In line with the nature of these activities, the importance of productivity in healthcare is invaluable, as healthcare needs shall always be promptly met. It is worth mentioning that the opposite of proactivity is passiveness, not reactiveness [87]. The passiveness issue is particularly pronounced in healthcare where untimely action expands and deepens the problem to incredibly large scales.

Within the healthcare context, risk is perceived as bold action taking and directing resources to seizing new opportunities with a certain probability of loss incurrence [14,17,88,105,116,117,118]. Under no circumstances shall entrepreneurial risk-taking in healthcare incur taking unreasonably high risk (similar to gambling). Still, healthcare is simply inseparable from risk, as each action of curement imposes risk. However, if one would choose not to start with the curative process, the risk might be even larger as certain diseases repeatedly occurred among more individuals and throughout different points of time. Hence, on the one hand, there is always the risk that the chosen method will not provide the expected and hoped outcomes, whereas on the other hand, taking no action might not only produce a negative outcome in that isolated case, but there is also a high probability this might lead to even larger consequences. As long as the risk-taking actions were done with deep care and in good faith, regardless of the specific outcome being positive or perhaps negative, the experience could serve for various other situations. Risk-taking will significantly depend on the autonomy of an organization but also the individual itself [119].

Though collaboration with other institutions is very important for a public entrepreneur [99], any kind of dependence on other organizations is undesirable, hence the focus should be on communication [120]. This makes it appropriate to take into account autonomy and independence the same way it is suggested for general entrepreneurship [87]. Actually, autonomy (including freedom and openness) is a key to developing public entrepreneurship [121]. It can be manifested through creating new autonomous or semi-autonomous units or enterprises [66], but also through independent individual activities in leading ideas to realization [112]. Autonomy varies as a function of size, managerial style and ownership structure, but also organization style and increased decentralization [87]. Most commonly, it is introduced through more decentralization and “flattening” organizational structure [122]. Autonomy in public healthcare enables isolation from political and similar influences, which increases responsibility but also gives freedom for resource allocation, which speeds up the process of decision-making, which is particularly important for entrepreneurship [29].

Competitive aggressiveness, or aggressive positioning towards competitors is very often used as a dimension of entrepreneurship [88,89,123]. It stands for specific response to threats from the environment [66,114]. Some contend it should not be practiced in non-profits, especially the public sector as public entrepreneurs are usually seeking for collaboration and communication opportunities with other peer organizations, rather than perceiving those as competitors [124]. Actually, very often public healthcare institutions jointly act in their attempts to provide health services. What they do is collaborate to fight the only competitor—illness and detrimental activities of the population. Hence, when talking about non/profits in healthcare, aimed at maximizing health, competitors are all those who prevent the institution in achieving its goals. Those could be producers of harmful products, causes of deviant behaviour, sources of health and safety risks, and the like.

As the literature supports each of the five dimensions, accounting for the aforementioned juxtaposed opinions in terms of how many entrepreneurial dimensions shall be taken into account, we decided to define two alternative specifications for each model. This way, in one of our specifications we observe innovation, proactivity, and risk-taking as the only three dimensions of entrepreneurship, whereas in the second specification we also add autonomy and competitive aggressiveness to the other three.

The next dilemma we faced is which approach for measuring entrepreneurship to take. We decided to account for what Petković and Sorak [125] emphasize, that is the coexistence of two approaches to studying entrepreneurship- single-perspective entrepreneurship and the multidimensional approach. Namely, according to the first approach, all entrepreneurial variables covariate, moving around roughly the same level, whereas the latter argues that variables are fully independent, meaning the difference in their representation might be very different.

Supporters of the single perspective approach deem dimensions of entrepreneurial orientations to covariate, meaning if an organization shows a high level of one variable, the other variables will gravitate to roughly the same level [126]. It was Miller [98] who first proposed the single-approach view. This was later supported by Covin and Slevin [14], who argue that a total level of entrepreneurship within an organization could be assessed by the three main dimensions, that is, a combination of the three. According to this approach, an organization fosters entrepreneurship to employ each of the entrepreneurial dimensions at the same time, asserting that each of the dimensions equally contributes to the entrepreneurial orientation. This implies that entrepreneurial orientation would only increase if all the dimensions simultaneously grow [127]. However, a number of scholars support the opposite, so-called multidimensional approach. As Arbaugh et al. [128] argue, research on dimensionality of entrepreneurship produced equivocal results. According to Arbaugh et al. [128], some authors supported the multidimensional approach [129,130], whereas others [131,132,133] supported the single-perspective approach. The main critique of the single-perspective approach comes from the argument that it is possible for each dimension to differently affect an organization’s performance [17]. Though, there are opinions [126] arguing that the single-perspective approach is generally preferred in a number of seminal studies of entrepreneurship [18,134,135,136,137]. Following Edmond and Wiklund [126], a main advantage of the single-perspective approach is being easier to apply analytically, and what is more, a number of studies proved a high level of correlation between the dimensions, implying that combining the variables in a single dimension might not alter empirical findings. Following the two approaches explained, we decided to introduce two more specifications for each of our models, so we could test whether the approach to measuring variables would alter empirical results. 

For the data collection and entrepreneurship estimation purposes, we noticed that research studies on this topic usually use interviews, expert talks [138], industry reports [139], national health associations’ surveys [29], as well as internal procedures review, such as memoranda, minutes, and correspondence [31]. Actually, most empirical studies of entrepreneurship use measurements based on self-evaluations. More precisely, they use questionnaires handed out to the observational units. Even studies dealing with attempts of defining entrepreneurship as a term use questionnaire surveys [140]. This also applies to research on entrepreneurship in healthcare and public entrepreneurship in general. Moreover, another technique used is interviews. However, these studies [6,31,62,138,139,141,142] mostly come down to asking managers (or at best also other employees) about their views of whether and how much entrepreneurship is being used in their organization. Beside the regular critiques on interviews as a method for collecting data on entrepreneurship, within healthcare it is particularly the case that employees might identify entrepreneurial orientations when they personally have them, or if they do not, they might not be even able to identify them [62]. Finally, healthcare entrepreneurship is quite difficult to measure, which is why questionnaires focus on what is simple and measurable, regardless of whether it fully reflects entrepreneurship (or management) [143]. 

There are a number of indices showing the level of entrepreneurship on a country level (such as, for example: Global Entrepreneurship Index—GEI, Regional Entrepreneurship Development Index—REDI, Total early-stage Entrepreneurial Activity—TEA, Entrepreneurial Employee Activity—EEA). Apart from standard critiques of indices, additional problems arise from the fact that these usually fail to grasp all the important features, then the data is being collected ad hoc, and they do not provide a representative picture [144]. Another caveat is that these indices (GEI, REDI, TEA) are not applicable as they do not consider intrapreneurship separately or, even if they do (for example, EEA), they do not provide industry- and sectoral-level data, which is why there is absolutely no available index specifically measuring intrapreneurship within the public healthcare sector. A number of authors, in their attempts to measure entrepreneurship as precisely as possible, developed different scales, such as, for example: High Entrepreneurship, Leadership, Professionalism Questionnaire—HELP-Q [112], Intrapreneurial Self-Capital Scale—ISCS [145], Psychological Capital Questionnaire—PCQ [146], Proactive Personality Scale—PPS [147], Rosenberg Self-Esteem Scale—RSES [148], Satisfaction With Life Scale—SWLS [149]. However, none of these are strictly related to public healthcare entrepreneurship. In terms of databases used, we noticed that other quantitative studies of entrepreneurship [29,150,151,152] mainly used EUROSTAT, GEM, REDI, OECD, WB, and WHO. Given this in addition to our previous mention of the WHO being the only organization monitoring public healthcare performance, we took advantage of the WHO database.

## 3. Materials and Methods

### 3.1. Sample

Our sample refers to almost the entire population of the world’s countries, that is, 192 members of the WHO. Covering a time span of 10 years, 2010–2019, with some missing data across countries for certain years, we constructed a strongly balanced panel. Despite various indices measuring entrepreneurship on a country level being available, there is nothing comparable for intrapreneurship, especially not for intrapreneurship within public healthcare. This being the only institution analysing public healthcare performance globally, we decide to use the WHO’s data. More precisely, the main observational data for this study was retrieved from the WHO’s Global Health Observatory database.

### 3.2. Model Specification

The first model we study in this paper can be represented as follows:

(1) $$hins_{it}=\beta_{0}+\beta_{1}dev\_lic_{it}+\beta_{2}dev\_lowmic_{it}+\beta_{3}dev\_upmic_{it}+\beta_{4}dev\_hic_{it}+\beta_{5}entrepreneurship_{it}+\epsilon_{it}$$

where *hins* stands for public healthcare institutions, index *it* indicates the country *i* (taking values from 1 to 192, depending on the country observed) in time period *t* (taking values from year 2010 to year 2019). Dummy variables *dev_lic*, *dev_lowmic*, *dev_upmic*, and *dev_hic* represent the level of development of an economy according to the classification accepted by the World Bank (that is, low-income, lower middle-income, upper middle-income, high-income). Finally, entrepreneurship indicates our variable of interest, that is, entrepreneurship being employed within the public health sector. Depending on the exact specification used, this can either be a single variable, or it is a vector of multiple dimensions of entrepreneurship.

The second model we study in this paper can be represented as follows:

(2) $$hpol_{it}=\beta_{0}+\beta_{1}dev\_lic_{it}+\beta_{2}dev\_lowmic_{it}+\beta_{3}dev\_upmic_{it}+\beta_{4}dev\_hic_{it}+\beta_{5}entrepreneurship_{it}+\epsilon_{it}$$

with *hpol* indicating public healthcare policies, whereas the other variables are same as in our first model.

For quantifying public healthcare institutions and policies, we refer to the WHO’s database. Within their extensive data, WHO provides specific assessment of quality of healthcare systems measured by the level of implementation of different aspects of International Health Regulations (IHR). IHR provides a comprehensive legal framework defining rights and obligations of countries in handling public health events and emergencies. These rules are legally binding for WHO member countries. Hence, each country is obliged to annually report on their compliance with the IHR. This dataset provides different scores (index values) the WHO assigns to a country depending on the level of development of the public healthcare system capacities and compliance with the IHR Framework. More specifically, the WHO sends a “States Parties Questionnaire” (also referred to as the IHR monitoring questionnaire) annually to the National IHR Focal Points for data collection. The questionnaire itself contains a checklist of 20 indicators, specifically designed for monitoring different capacities of a public healthcare system. The data is further processed into indices indicating the proportion of attributes (a set of elements of functions reflecting the level of performance) being attained. In this way, the WHO assigns a score on a scale of 0 to 100 to each country for various categories (indicators) depending on the level of compliance with the IHR requirements. Thereby, 0 stands for a complete absence of compliance, whereas 100 denotes the full compliance with a certain IHR criteria. Looking at capacities that IHR measures, we notice that among others, they measure characteristics of a national healthcare system that closely fit the definition of public healthcare institutions and public healthcare policies (as defined in Section 2). We decide to use this data for our dependent variables. As we assess institutions using the *preparedness score*, 0 would mean a country completely deviates, whereas 100 suggests a country fully complies with preparedness requirements that read as follows (see https://www.who.int/data/gho/indicator-metadata-registry/imr-details/4411) (accessed on 22 December 2021): “Preparedness includes the development of national, intermediate and local community/primary response level public health emergency response plans for relevant biological, chemical, radiological and nuclear hazards. Other components of preparedness include mapping of potential hazards and hazard sites, the identification of available resources, the development of appropriate national stockpiles of resources and the capacity to support operations at the intermediate and local community/primary response levels during a public health emergency”. 

Likewise, we assess policies using the *legislation score*. With respect to this, a score of 0 would suggest a country completely deviates, whereas 100 suggests a country fully complies with legislation requirements that read as follows (see https://apps.who.int/gho/indicators/imr.jsp?id=4407) (accessed on 22 December 2021): “An adequate and appropriate legal framework to support and enable implementation of the IHR is needed within States Parties. Policies which identify national structures and responsibilities as well as the allocation of adequate budgets are also important”.

A comprehensive assessment (i.e., measurement) of the public healthcare sector performance would involve measuring the population health, public healthcare services, and *public healthcare infrastructure* [152]. Each of these categories formally consists of a number of elements, although not all of the elements are measurable (or amenable to measurement). Namely, it is usually the case that the physical observation, recording, or quantifying of these elements is not feasible at all [60]. What is more, even when measuring is feasible, it is the question of ethics that usually prevents performing this process. Another issue complicating the assessment of the public healthcare sector is the limited access to databases containing information of interest (e.g., sometimes certain data is only available for the purposes of internal use within a country). Hence, there is a general agreement that easy-to-use instruments for measuring the effects or quality of public healthcare do not exist [2].

For measuring public healthcare infrastructure, some commonly used variables are government expenditure, that is, fiscal expenses for this category [67]. However, a general caveat of such measures is that they reflect overall capital investments during a fiscal year. This means such variables suggest a flow rather than stock (that is, a change in the stock, and not the level of the stock). Moreover, they reflect changes in tangible infrastructure without taking into account intangible infrastructure. Hence, we follow Hall and Jones [4] in their approach to measuring infrastructure through institutions and policies. 

Many scholars contend that for assessing the quality of the public healthcare sector, one must not observe a system of a particular country in isolation but should rather compare the outcomes within a country to some general country specifics (such as population), and also should compare the outcomes across countries [153,154]. Particularly, when measuring public healthcare policies, one should assess if the goals are politically correspondent (meaning the goals of public healthcare should correspond to the other goals of the country but also to the healthcare goals of other countries) [155].

Following recommendations from other authors, that in order to measure the quality of a certain healthcare system one should make relative comparisons, we deemed IHR appropriate as they not only provide a relative comparison across countries, but also compare each national healthcare system to a certain benchmark (that is, IHR is used as a benchmark and hence uses scores from 0 to 100).

Oxford Dictionary defines quality as “the standard of something when it is compared to other things like it; how good or bad something is”. As WHO developed IHR as a form of standards that should be followed in order for countries to achieve their common goals in preserving public health, we assume that one way to estimate the quality of a certain public healthcare system is to assess how good they are in meeting these standards. 

When gathering data for our independent variable, as explained in the literature review, a main caveat with researching entrepreneurship within the public healthcare sector is a general lack of precise data. None of the databases on entrepreneurship provide data on a sectoral level (that is, entrepreneurship in the healthcare sector precisely), nor data on entrepreneurship within the public sector. Following our argument from the literature review, where we provided an overview of three approaches to measuring entrepreneurship arguing that the effect or performance-based approach provides the least bias, we decided to find data on the effects of entrepreneurship within the public healthcare system. For these purposes, we again refer to the WHO database, given this is the only comprehensive database on healthcare systems around the globe. The IHR dataset provides information on further characteristics of national healthcare systems; that is, their performance in different areas. Within these indicators, we were able to identify indicators that closely match to the potential effects of entrepreneurship, when entrepreneurship is observed through different dimensions, as explained in the literature review. That is, within IHR indicators, we were able to identify variables that qualify for a very good approximation of innovativeness, proactivity, risk-taking, autonomy, and competitive aggressiveness within the public healthcare sector (see https://www.who.int/data/gho/indicator-metadata-registry/) (accessed 22 December 2021). Hence, we decided to use this dataset for quantification of our independent variables. 

To summarize, we measure variables from our model combining the WHO Global Health Observatory and WB data. More specifically, for our dependent variables, PHI, and endogenous independent variables (entrepreneurship), we used the International Health Regulations’ core capacity index data. For the independent exogenous variables (a country’s level of development), we used the WB data on the level of economic development, that is, the WB classification of countries depending on their level of development throughout the years observed.

Thus, explanatory variables in our model are approximated as follows. Variable Public healthcare institutions (*hins*) denotes the preparedness score (index measuring the level of development of national, subnational, and local response plans, as well as strategies aimed at combating various hazards; this index also includes the level of mapping of the potential hazards, identification of available resources, as well as the development of adequate national stocks of resources and capacities to support activities within the community in the face of an emergency). Variable Public healthcare policies (*hpol*) denotes a legislation score (defined as a wide range of legal, administrative, and other government instruments used in the capacity building processes regarding public health; harmonization of the aforementioned legislation with international health regulation prescribing in detail what is needed for achieving a higher quality public healthcare system).

Variables of interest are approximated as follows. Variable Innovativeness (*inno*) denotes a laboratory score, defined as the extent to which the system responds to the alerts, that is the extent to which problems are detected, studied, and addressed, including the laboratory sampling, analysis, and research needed to be performed in order to create a single solution to a problem. Variable Proactivity (*proa*) denotes a surveillance score, which is the index capturing the level of how rapid the detection of risks in healthcare is, as well as the existence of a prompt assessment, notification, and preparation of responses to the identified risks. Variable Risk-taking (*risk*) denotes a risk-communication score, defined as the existence of a complex system allowing for risks to be clearly defined, threats identified, the vulnerability of the system to be assessed, and the resilience to risk promoted; accordingly, it also measures the capacity to deal with risks and emergencies in public health. Variable Autonomy (*auto*) denotes a coordination score (a higher value of this score is assigned to health systems in which the implementation of the necessary activities is performed while simultaneously involving multiple hierarchical levels, followed with the simultaneous coordination). Variable Competitive aggressiveness (*comp*) denotes a response score (indicating the level of command, communication, and control mechanisms needed to coordinate and manage epidemics and other significant events in the public sector; the country is assigned a higher value of this index if it has provided teams available to provide a rapid response 24/7; this index also takes into account emergency management, infection control, and decontamination capacities).

Control variables are measured by assigning a binary choice outcome of 1 if a country was classified within the specific group, with groups being: low-income economy, lower middle-income economy, higher middle-income economy, and high-income economy.

Accounting for missing values resulting from the unavailability of data for certain countries for a specific year, we are left with 1484 observations in total. Table 1 below gives summary statistics for the variables used in our model. Since we use indices as proxies for each of the variables in our model, expectedly, their values range from 0 to 100. Interestingly, though all our variables being indicators measured within a single survey, contrary to what one would normally expect, they do not seem to be completely comoving. Namely, dispersion of a dataset relative to its mean seems to differ substantially across the variables observed.

To check for potential multicollinearity, we estimate Pearson’s correlation coefficients amongst the independent variables. The correlations are overall low to moderate, suggesting multicollinearity is unlikely to occur. These results are not reported here, but are available upon request. The independent variables were not expected to suffer from collinearity as the proxies we used are indicators that the WHO takes together and adds a couple more in order to obtain a composite level of international compliance with the International Health Regulations. Hence, it is unlikely to believe that the WHO would count the same things twice or more, as this would contradict the general framework of the International Health Regulations.

### 3.3. Empirical Strategy

Recognizing difficulties in assessing the performance of non-profits, including public healthcare institutions, some authors argue that, regardless of the exact evaluation method used, one useful thing would be comparing the individual performance with their peer group [124]. Such an argument makes it sensible to think that a cross-sectional study, or even more, one with multiple cross sections—that is, panel observations—would be appropriate. We followed recommendations from other studies [156,157,158], and decided to use a panel data setting, specifically choosing the fixed effects (FE) estimator. An advantage of the FE estimator is that it controls for systemic differences across groups and performs a quasi-experiment that enables the observation of actual effects, isolated from “noise” [159,160]. One of the reasons why other scholars (e.g., [161]) recommend using fixed effect is because it deals with the endogeneity issue in terms of independent variable being correlated with a part of residual that strictly depends on the variable (i.e., a country in this study) being fixed. That is, the fixed effect observes residual or the error term as a composite variable made of an idiosyncratic part and another, time-invariant part. In contrast, the random effects (RE) estimator relies on a rigid assumption of no correlation between unobserved effects and independent variables. Although the RE model is based on this rigid assumption, we conducted the Hausman test for each model to determine whether the FE or RE models are preferred [162,163]. For each model, the Hausman tests indicate that the FE models are preferred. These results are not reported here, but are available upon request.

In defining the PHE, we refer to seminal papers by Morris and Jones [39], who posit that the underlying dimensions of entrepreneurship are congruent regardless of the context. Building up on this, we follow the general approach deeming entrepreneurship as an amalgam of innovativeness, risk-taking, and proactivity [14,87,127,164]. Furthermore, we examine an alternative specification following alternative explanations of entrepreneurship, taking into account both autonomy [165,166,167], as well as competitive aggressiveness [17,168] on the top of the first three dimensions. This being added to the earlier explained discussion on choosing between the single-perspective versus multidimensional approach to studying entrepreneurship, we ended up having four specifications for each of our models. Depending on how we observe entrepreneurship, our four specifications are as follows: (i) entrepreneurship being measured as a composite index of three dimensions—innovativeness, proactivity, and risk-taking; (ii) entrepreneurship measured as a composite index of five dimensions—innovativeness, proactivity, risk-taking, autonomy, and competitive aggressiveness; (iii) entrepreneurship being measured through three separate dimensions—innovativeness, proactivity, and risk-taking; (iv) entrepreneurship measured through five separate dimensions—innovativeness, proactivity, risk-taking, autonomy, and competitive aggressiveness.

For the specifications following the single perspective approach, we follow Azami [6], who suggests the use of composite indices. Here again, as explained in Section 2, we decided to account for both general views on entrepreneurship, with one of them contending entrepreneurship has three dimensions—innovativeness, risk-taking, and proactivity [14,87,127,164]—which are captured by the variable *entre3* and thus reflect the view that entrepreneurship consists of three comoving dimensions. Further, we accounted for the other theoretical position, claiming that entrepreneurship has more than three dimensions. More specifically, we followed scholars who identified autonomy [165,166,167] and competitive aggressiveness [17,168] as the fourth and fifth indispensable entrepreneurial dimensions. That way, we composed the variable *entre5*, which reflected the view that entrepreneurship consisted of five comoving dimensions—innovativeness, risk-taking, proactivity, autonomy, and competitive aggressiveness.

Hence, *entre3* stands for the composite index of innovation, proactivity, and risk-taking, whereas *entre5* is the composite index of these three dimensions together with autonomy and competitive aggressiveness. Variables *entre3* and *entre5* correspond to the three and five dimensions of entrepreneurship, respectively, when being observed separately, as suggested by the multidimensional approach to studying entrepreneurship.

## 4. Results

Table 2 shows results for the first dependent variable, testing whether and how much improving entrepreneurship alters the quality of public health institutions. Overall, the positive relationship is established at the 1% level of significance (except for the variable *inno* in Model 4). Interestingly, in none of the model specifications did the level of development of a country have a significant effect. This means that countries do not benefit from an increase in the quality of public institutions as they move from one to another level of development.

Model 1 shows that a unit increase in the average score of entrepreneurship represented as a combination of innovation, proactivity, and risk-taking, increases the score of quality of health institutions by 0.749. When autonomy and competitive aggressiveness are added to the other three (Model 2), it turns out that unit increase in average score of entrepreneurship has even higher impact; that is, it increases the score of quality of health institutions by 0.909. In order to determine if these apparent differences are statistically significant, we ran a t-test on the difference in means and found that differences between the coefficients on the variables *entre3* and *entre5* in Models 1 and 2 are statistically significant at the 1% level (*p* < 0.01).

Model 3 follows a multidimensional approach to measuring entrepreneurship and thus separately observes the impact of increasing innovation, proactivity, and risk-taking separately on health institutions. The coefficients for each of these independent variables are highly statistically significant at the 1% significance level (*p* < 0.01), while their values are 0.167 for innovation, 0.24 for proactivity, and 0.319 for risk-taking, respectively. 

Finally, Model 4, that takes into account separately all five dimensions of entrepreneurship, shows that four dimensions (proactivity, risk taking, autonomy, and competitive aggressiveness) are highly statistically significant at the 1% level (*p* < 0.01), while the innovation dimension is not statistically significant at any conventional level. 

Table 3 shows results for the second dependent variable, testing whether and how much improving entrepreneurship affects the quality of public health policies. The results are similar to those from the first model, though they show a slightly lower economic significance. That is, these results suggest entrepreneurship produces effects to health institutions of a slightly lower magnitude compared to the effects on health institutions. 

Model 5 shows a positive correlation between entrepreneurship and health policies at the 1% level of statistical significance (*p* < 0.01). It shows that a unit increase in the average score of entrepreneurship, represented as a combination of innovation, proactivity, and risk-taking, increases the score of quality of health policies by 0.63. Adding autonomy and competitiveness and then taking their average (Model 6), shows at the 1% significance level (*p* < 0.01) that a unit increase in the average score of entrepreneurship has even higher impact; that is, if the average of five dimensions of entrepreneurship increases by one unit, the score of quality of health policies increases by 0.809. When the t-test is performed, we can conclude that the difference between the coefficients on the variables *entre3* and *entre5* in Models 5 and 6 are statistically significant at the 1% level (*p* < 0.01).

Moving to Model 7 where we measure entrepreneurship through its three main entrepreneurship dimensions separately, results suggest a positive correlation between each of the dimensions and the quality of health policies. The coefficients take values of 0.197 for innovation, 0.251 for proactivity, and 0.193 for risk-taking. At last, Model 8, that takes into account separately all five dimensions of entrepreneurship, produces results of a mixed statistical significance, depending on the dimension. The coefficients take values of 0.135 for innovation (*p* < 0.05), 0.133 for proactivity (*p* < 0.05), 0.101 for risk-taking (*p* < 0.05), 0.303 for autonomy (*p* < 0.01), and 0.125 for competitive aggressiveness (*p* < 0.1). 

## 5. Discussion

Overall, our empirical results, though economically modest, show a positive and statistically significant correlation between public healthcare entrepreneurship, as an independent variable, and public healthcare infrastructure, as a dependent variable. When comparing the results, it seems that entrepreneurship produces a somewhat larger effect on quality of health institutions, relative to health policies.

However, when comparing results from different specifications within a model, we revealed some interesting findings. Namely, measuring entrepreneurship as a composite score of five variables compared to three variables produces statistically different results. According to our findings from both models, adding autonomy and competitive aggressiveness to innovation, proactivity, and risk-taking provides a better explanation of the model and additionally shows that the effects of the “additional two dimensions” are not negligible. Such results, at least to some extent, complement the ongoing literature discussion on whether the three “main” dimensions capture most of entrepreneurship or the model should be extended by taking into account the additional two dimensions.

In the next step, we tested whether the individual coefficients obtained for each of the dimensions differed among each other. In Models 3 and 4, we found no support that coefficients are qualitatively the same. That is, our test either proved a statistically significant difference between their magnitudes or produced somewhat ambiguous results. Interestingly, when performing the same tests for models in Table 3, we came to mixed conclusions. Overall, the difference between magnitudes of individual dimensions was generally statistically significant or ambiguous with some exceptions. Namely, the results obtained in Model 7 show no difference between magnitudes of innovativeness and risk-taking. That is, when entrepreneurship is observed through the three dimensions, innovativeness and risk-taking produce the same qualitative effects to the quality of public health care policies, ceteris paribus. Similarly, when performing the same test for the coefficients obtained in Model 8, we find that magnitudes of innovativeness, proactivity, and competitive aggressiveness do not differ, that is, these three dimensions ceteris paribus produce the same qualitative effects as public healthcare policies, when entrepreneurship being observed through five dimensions. Putting these results together, we conclude that we find no strict support to any of the theories on dimensionality of entrepreneurship. Apparently, the nature of movement of entrepreneurial dimensions possibly depends on the context, particularly on the outcome variable observed. In some situations, dimensions tend to covariate, making the case for the single-dimension approach, whereas in other situations all the dimensions behave differently, supporting the multidimensional approach to measuring entrepreneurship.

To our surprise, the level of the development of a country showed no impact on the quality of PHI. Such results might produce interesting policy implications. Namely, given that the quality of PHI is not contingent upon the level of the development of an economy, this additionally stresses the importance of research similar to what we have carried out here. This means that the quality of PHI shall not suffer from the country-specifics, meaning countries with different levels of development may use entrepreneurship to “level the playing field”. That is, being a high-income economy does not guarantee a high quality of PHI, same as being a low-income economy shall not be an impediment to the development of PHI, per se.

Furthermore, the overall quality of PHI depends on many other variables. However, the main question is whether the remaining drivers of variance in and level of PHI quality are endogenous or exogenous. Despite not being reported, we run a regression of all our specifications for each of the two models with an alternative control variable being the geographical region of a country instead of its level of development. We opted for this control variable referring to a range of research [69,71,142,169,170,171,172] recognizing the dependency of entrepreneurship on the regional environment, arguing that certain regions have better predispositions for fostering entrepreneurship. We extended these assumptions with findings from other research [4,73,173] explaining that the institutional framework is dependent on the geographical region, as this is closely connected to occupations and settlements throughout history. Finally, we took into account studies showing how institutional environment influenced entrepreneurship [75,166,174,175]. To our surprise, we found that the quality of PHI was in no way dependent on the geographical region of a country. Again, though surprising, these results have similarly encouraging policy implications as is the case with the level of development. Actually, given that the country can affect their level of development by maintaining economic growth, whereas a country’s region is completely exogenous both in the short, as well as long term and even perpetually, knowing that the quality of the PHI is not contingent upon such country-specifics, implies even more impetus to developing intrapreneurship within the public healthcare sector.

Summing up, we conclude there is not enough evidence to disregard the positive effects that public healthcare entrepreneurship has on the quality of public healthcare infrastructure. Proving the PHI to be independent of several exogenous country-specifics additionally underpins our suggestions for policy makers to become strongly involved in supporting the development of the PHE. This can be followed by policy recommendations from other studies; namely, for developing internal entrepreneurship in healthcare, internal organizational factors are particularly significant and they should enable independence, support, rewards, flexibility, and organizational procedures that would support employees in healthcare in their intents to foster entrepreneurial spirit [62,176,177,178,179]. As much as the institutional environment affects entrepreneurship [180,181], so does entrepreneurship the other way round [182].

## 6. Conclusions

This study explores the effects of entrepreneurship within public healthcare (PHE) on the quality of the public healthcare infrastructure (PHI) using the sample of 192 countries being observed through the period of 2010 to 2019. Regardless of choosing health policies or health institutions as approximators of PHI, we found statistically significant evidence of its quality being influenced by the level of PHE. 

For each of the models, the first having public healthcare institutions and the second having public healthcare policies as a dependent variable, we defined four model specifications. We derived these four specifications combining juxtaposed stances in the literature on two questions. The first is a question of how many dimensions shall be included when measuring entrepreneurship; that is, whether innovation, proactivity, and risk-taking provide a substantial explanation of entrepreneurship, or some other dimensions should be added to these three, such as autonomy and competitive aggressiveness. The second field of disagreement within the literature on entrepreneurship concerns the approaches to measuring entrepreneurship, with one stance being that the dimensions of entrepreneurship covariate, whereas the confronting one claims that they move independently. Overall, we find that adding two more dimensions to the generally adopted three provides for a better capturing of entrepreneurship. In terms of the effects each separate variable of PHE produces to the quality of the PHI, we show that the main three dimensions generally produce the same effects. However, in terms of the additional two dimensions, empirical results show that competitive aggressiveness produces larger effects on public healthcare institutions, while producing qualitatively the same effects as the other variables do on public healthcare policies. Conversely, while autonomy produces similar effects as other variables on the quality of public healthcare institutions, it produces the largest effects on the public healthcare policies.

Regarding the question of dimensionality of entrepreneurship, for the models examining the effects of entrepreneurship dimensions on public healthcare institutions we found no support for the single-dimensional approach and its general presumption that all the dimensions tend to covariate (ceteris paribus produce the same effects as the outcome variable), but in some cases we did find support for the multidimensional approach. Interestingly, when we tested the coefficients obtained in models that estimated the effects of entrepreneurship dimensions on public healthcare policies, we found that some dimensions tended to covariate, whereas others did not. This means we find support for both the multidimension as well as the single-dimension approaches. Given the ambiguity of these results, we finally conclude to take no strict position on dimensionality of entrepreneurship. That is, we deem that dimensionality of entrepreneurship (in terms of a question whether individual dimensions ceteris paribus produce the same or different effects to the outcome variable) depends on the outcome variable observed.

Such results not only support our main argument that PHE positively affects PHI, but provides additional findings regarding the discussion in the literature on the number of entrepreneurial dimensions, as well as the approaches to measuring entrepreneurship, providing by this a contribution to the broad literature on entrepreneurship. In support of this position, we add some other authors’ (e.g., [181]) assertions of lacking research on applying entrepreneurship to a rather complex healthcare environment.

Contrary to the literature standings, PHI proved to be independent of country specifics, such as level of the development or the geographical region of a country. Despite seeming discouraging in the manner of having left a larger portion of variation in and the size of PHI quality being unexplained, these results have significant implications. Namely, this would imply that a country’s quality of PHI should not suffer, nor benefit from the inherited country specifics; that is, from the variables exogenous in the short term (such as the level of a country’s development), not the perpetually exogenous variables (such as a geographical positioning).

From a pragmatic point of view, the results of this study provide several practical implications that might inform policymakers and public healthcare organizations in designing rules and programmes to improve healthcare development. For example, governments (especially ministries of health) may use findings from this study as a guideline for promoting entrepreneurship within public healthcare organizations. This could be provided through the combination of issuing internal rules and procedures for stimulating entrepreneurial behaviour among healthcare employees, but also through providing grants, fundings, and different awards to support entrepreneurial ventures within the public healthcare sector. Similarly, the results of this study might be useful for managers of public healthcare organizations, especially when making decisions about resource allocation. Knowing that entrepreneurship provides an increase in the quality of public healthcare’s intangible infrastructure might incentivize them to allocate more resources to support entrepreneurial behaviour within their organizations. Furthermore, the underlying relationship established in this study essentially suggests that countries benefit from a better compliance with the international health regulations. This might serve as an incentive for national healthcare systems to put more effort into providing better compliance with the IHR. Likewise, these results could also be useful for the WHO in re-evaluating the list of 20 indicators used and perhaps considering introducing further indicators of public healthcare development. Ultimately, the WHO should also think about rescaling the indices used. With extending the regulations, they could encourage countries to constantly improve public healthcare services provision.

Taking into account the ongoing global pandemic, the policy implications of our study become even more pronounced. Namely, as some authors warn, increased international mobility and trade pose a risk to disease spread and the outbreak of pandemics [183]. Thus, providing a sufficient quality of healthcare infrastructure would help create positive externalities perhaps even beyond a country’s borders. Likewise, any failures in the healthcare system of a country could generate negative externalities and negative spillovers way beyond their national borders. 

Finally, there are several limitations of our study that can serve as suggestions for future studies. First, with our research subject being quite narrow—studying intrapreneurship within public healthcare—a significant opus of comparable research is not available. As mentioned, there is no available database strictly measuring entrepreneurship within public healthcare. However, as mentioned within the limitation of studies observing entrepreneurship, using proxy variables might be a better solution. Second, another caveat arises from the type of variables used. Namely, a natural feature of scores and count variables is a question of ranking on the scale. Simply put, if a country already achieves a score of 100, which would imply a full compliance to the International Health Regulations, would this imply that it cannot progress any further, or regardless of the progress achieved, its score would be capped at 100. Finally, as we mention in the literature review, any kind of self-reported data is susceptible to bias. Hence, we warn that data for IHR is reported by the national health institutes to the WHO, which might suggest a certain degree of subjectivity while reporting the data. However, as explained throughout the paper, we used proxy variables. This means while doing the self assessment, the NHIs were not aware they are essentially rating entrepreneurial variables. Thus, the bias might only be the case if they misrated a certain component of their institution, which is indirectly translated to what we measure as entrepreneurship. Last but not least, the WHO itself significantly controls the bias by providing a unified form together with detailed instructions on how the IHR questionnaires shall be filled out. For this reason, we believe the data WHO reproduces are substantially reliable. These limitations, though being beyond the reach of our influence, might serve as a potential motivation for future studies on this or the related topics.

## Figures and Tables

**Table 1:** Variable description and summary statistics.

Variables	Variable Description	Mean (Standard Deviation)
*hpol*	Variable approximating public healthcare policies, which is the index ranging from 0 to 100	69.93 (32.71)
*hins*	Variable approximating public healthcare institutions, which is the index ranging from 0 to 100	64.36 (29.62)
*dev_lic*	Dummy variable (DV)= 1 if a country being classified by the WB as a low-income economy in the year observed; zero otherwise	0.17 (0.38)
*dev_lowmic*	DV = 1 if a country being classified by the WB as a lower middle-income economy in the year observed; zero otherwise	0.25 (0.44)
*dev_upmic*	DV = 1 if a country being classified by the WB as a higher middle-income economy in the year observed; zero otherwise	0.29 (0.45)
*dev_hic*	DV= 1 if a country being classified by the WB as a high-income economy in the year observed; zero otherwise	0.29 (0.45)
*inno*	Variable approximating public healthcare innovation as an entrepreneurship dimension, which is the index ranging from 0 to 100	75.89 (22.88)
*proa*	Variable approximating public healthcare proactivity as an entrepreneurship dimension, which is the index ranging from 0 to 100	78.82 (19.77)
*risk*	Variable approximating public healthcare risk-taking as an entrepreneurship dimension, which si the index ranging from 0 to 100	69.30 (28.52)
*auto*	Variable approximating public healthcare autonomy as an entrepreneurship dimension, which is the index ranging from 0 to 100	74.18 (25.16)
*comp*	Variable approximating public healthcare competitive aggressiveness as an entrepreneurship dimension, which is the index ranging from 0 to 100	74.75 (23.60)
*entre3*	Variable approximating public healthcare entrepreneurship as an arithmetic mean of the variables *inno*, *proa*, and *risk* (measuring three entrepreneurship dimensions)	74.67 (19.70)
*entre5*	Variable approximating public healthcare entrepreneurship as an arithmetic mean of the variables *inno*, *proa*, *risk*, *auto*, and *comp* (measuring five entrepreneurship dimensions)	74.59 (19.46)

**Table 2:** Empirical results for the first dependent variable—*hins* (public healthcare institutions).

Variables	Model 1	Model 2	Model 3	Model 4
*dev_lic*	4.598	2.071	3.904	−0.892
	(7.946)	(7.683)	(7.741)	(7.423)
*dev_lowmic*	3.024	1.517	2.653	0.259
	(6.427)	(6.410)	(6.394)	(6.407)
*dev_upmic*	−1.647	−1.231	−1.636	−1.310
	(4.559)	(3.912)	(4.164)	(3.537)
*Inno*			0.167 ***	0.071
			(0.041)	(0.043)
*Proa*			0.240 ***	0.140 ***
			(0.045)	(0.044)
*Risk*			0.319 ***	0.214 ***
			(0.034)	(0.033)
*entre3*	0.749 ***			
	(0.048)			
*entre5*		0.909 ***		
		(0.049)		
*Auto*				0.112 ***
				(0.033)
*Comp*				0.366 ***
				(0.045)
*Constant*	−0.845	−11.809 **	1.110	−10.715 **
	(4.927)	(4.910)	(5.071)	(5.112)
No of obs.	1482	1482	1482	1482
R^2^	0.364	0.421	0.370	0.440

Notes: Robust standard errors in parentheses: *** *p* < 0.01; ** *p* < 0.05.

**Table 3:** Empirical results for the second dependent variable—*hpol* (public healthcare policies).

Variables	Model 5	Model 6	Model 7	Model 8
*dev_lic*	−5.376	−7.822	−5.361	−6.845
	(10.710)	(10.058)	(10.787)	(10.242)
*dev_lowmic*	−3.772	−5.115	−3.613	−5.118
	(8.829)	(8.271)	(8.924)	(8.531)
*dev_upmic*	−4.188	−3.745	−4.067	−3.623
	(8.107)	(7.519)	(8.165)	(7.734)
*Inno*			0.197 ***	0.135 **
			(0.054)	(0.054)
*Proa*			0.251 ***	0.133 **
			(0.058)	(0.054)
*Risk*			0.193 ***	0.101 **
			(0.045)	(0.046)
*entre3*	0.630 ***			
	(0.067)			
*entre5*		0.809 ***		
		(0.071)		
*Auto*				0.303 ***
				(0.051)
*Comp*				0.125 *
				(0.065)
*Constant*	19.624 ***	7.580	18.824 **	7.521
	(7.452)	(7.701)	(7.814)	(8.104)
No of obs.	1482	1482	1482	1482
R^2^	0.227	0.273	0.228	0.284

Notes: Robust standard errors in parentheses: *** *p* < 0.01; ** *p* < 0.05; * *p* < 0.1.

## Data Availability

The data used in the study are from the WHO Global Health Observatory and the World Bank.

## References

[1] Insecurity Insight (2021). Violence against Health Care during the COVID-19 Pandemic in 2020.

[2] Scholz S., Ngoli B., Flessa S. (2015). Rapid assessment of infrastructure of primary health care facilities—A relevant instrument for healh care systems management. BMC Health Serv. Res..

[3] Lavinghouze S.R., Snyder K., Rieker P.P. (2014). The component model of infrastructure: A practical approach to understanding public health program infrastructure. Am. J. Public Health.

[4] Hall R.E., Jones C.I. (1999). Why do some countries produce so much more output per worker than others?. Quart. J. Econ..

[5] Nambisan S. (2015). Embracing Entrepreneurship Across Disciplines: Ideas and Insights from Engineering, Science, Medicine and Arts.

[6] Azami S. (2013). Intrapreneurship “an exigent employment”. Intern. J. Scien. Tech. Res..

[7] Hinz V., Ingerfurth S. (2013). Does Ownership Matter Under Challenging Conditions?: On the relationship between organizational entrepreneurship and performance in the healthcare sector. Public Manag. Rev..

[8] Koelewijn W.T., Ehrenhard M.L., Groen A.J., Van Harten W.H. (2012). Intra-oragnizational dynamics as drivers of entrepreneurship among physicians and managers in hospitals of western countries. Soc. Sci. Med..

[9] Ratten V. (2012). A theoretical framework of entrepreneurship and innovation in healthcare organisations. Intern. J. Soc. Entrep. Innov..

[10] Pérez-Luño A., Wiklund J., Cabrera R.V. (2011). The dual nature of innovative activity: How entrepreneurial orientation influences innovation generation and adoption. J. Bus. Vent..

[11] Hernández D., Carrión D., Perotte A., Fullilove R. (2014). Public health entrepreneurs: Training the next generation of public health innovators. Public Health Rep..

[12] Dehe B., Bamford D. (2017). Quality Function Deployment and operational design decisions–a healthcare infrastructure development case study. Prod. Plan. Control.

[13] Pereira K., Peters R., Lallemand N., Zuckerman S. (2017). Puerto Rico Health Care Infrastructure Assessment. Research Report. https://www.urban.org/sites/default/files/publication/87011/2001050-puerto-rico-health-care-infratructure-assessment-site-visit-report_1.pdf.

[14] Covin J.G., Slevin D.P. (1989). Strategic management of small firms in hostile and benign environments. Strat. Manag. J..

[15] Naman J.L., Slevin D.P. (1993). Entrepreneurship and the concept of fit: A model and empirical tests. Strat. Manag. J..

[16] Wiklund J., Shepherd D. (2003). Knowledge-based resources, entrepreneurial orientation, and the performance of small and medium-sized businesses. Strat. Manag. J..

[17] Lumpkin G.T., Dess G.G. (2001). Linking two dimensions of entrepreneurial orientation to firm performance: The moderating role of environment and industry life cycle. J. Bus. Vent..

[18] Covin J.G., Green K., Slevin D.P. (2006). Strategic Process Effects on the Entrepreneurial Orientation—Sales Growth Rate Relationship. Entrep. Theory Pract..

[19] Wiseman V., Guiness L., Wiseman V. (2011). Key concepts in health economics. Understanding Public Health—Introduction to Health Economics.

[20] Bassanini A., Scarpetta S. (2002). The Driving Forces of Economic Growth.

[21] Bishai D., Sherry M., Pereira C.C., Chicumbe S., Mbofana F., Boore A., Smith M., Nhambi L., Borse N.N. (2016). Development and usefulness of a district health system tool for performance improvement in essential public health functions in Botswana and Mozambique. J. Pub. Health Manag. Pract..

[22] Bloland P., Simone P., Burkholder B., Slutsker L., De Cock K.M. (2012). The role of public health institutions in global health system strengthening efforts: The US CDC’s perspective. PLoS Med..

[23] Reeves A., McKee M., Basu S., Stuckler D. (2014). The political economy of austerity and healthcare: Cross-national analysis of expenditure changes in 27 European nations 1995–2011. Health Policy.

[24] De Costa A., Diwan V. (2007). Where is the public health sector?: Public and private sector healthcare provision in Madhya Pradesh, India. Health Policy.

[25] WHO (2007). The World Health Report 2007: A Safer Future—Global Public Health Security in the 21st Century.

[26] Ruger J.P. (2005). The changing role of the World Bank in global health. Am. J. Pub. Health.

[27] Slavin S. (1996). Macroeconomics. The McGraw-Hill Series. Economics.

[28] Głód G. (2015). Measurement of Public Entrepreneurship in the Polish Public Sector. Managing Intellectual Capital and Innovation for Sustaimable and Inclusive Society.

[29] Jacobson P.D., Wasserman J., Wu H.W., Lauer J.R. (2015). Assessing entrepreneurship in governmental public health. Am. J. Pub. Health.

[30] Vargas-Halabí T., Mora-Esquivel R., Siles B. (2017). Intrapreneurial competencies: Development and validation of a measurement scale. Eur. J. Manag. Bus. Econ..

[31] Maguire S., Hardy C., Lawrence T.B. (2004). Institutional entrepreneurship in emerging fields: HIV/AIDS treatment advocacy in Canada. Acad. Manag. J..

[32] Roberts N.C., King P.J. (1996). Transforming Public Policy: Dynamics of Policy Entrepreneurship and Innovation.

[33] Klein P.G., Mahoney J.T., McGahan A.M., Pitelis C.N. (2010). Toward a theory of public entrepreneurship. Euro. Manag. Rev..

[34] Kearney C., Hisrich R., Roche F. (2008). A conceptual model of public sector corporate entrepreneurship. Intern. Entrep. Manag. J..

[35] Bernier L., Hafsi T. (2007). The changing nature of public entrepreneurship. Pub. Adm. Rev..

[36] Smallbone D. (2008). Entrepreneurship and Small Business Development in Post-Socialist Economies.

[37] Legge J.M., Hindle K.G. (2004). Entrepreneurship: Context, Vision and Planning.

[38] Yu T. (1997). Entrepreneurial state: The role of government in the economic development of the Asian newly industrialising economies. Develop. Policy Rev..

[39] Morris M.H., Jones F.F. (1999). Entrepreneurship in established oranization: The case of the public sector. Entrep. Theory Pract..

[40] Roberts N.C. (1992). Public entrepreneurship and innovation: Introduction. Pub. Product. Manag. Rev..

[41] Lewis E., Hoover J.E., Moses R., Rickover H.G. (1980). Public Entrepreneurship: Toward a Theory of Bureaucratic Political Power.

[42] Lindholst A.C. (2019). Addressing public-value failure: Remunicipalization as acts of public entrepreneurship. J. Econ. Policy Reform.

[43] Tremml T. (2019). Linking two worlds? Entrepreneurial orientation in public enterprises: A systematic review and research agenda. Ann. Public Coop. Econ..

[44] Paik Y., Kang S., Seamans R. (2019). Entrepreneurship, innovation, and political competition: How the public sector helps the sharing economy create value. Strat. Manag. J..

[45] Leyden D.P., Link A.N. (2015). Public Sector Entrepreneurship: US Technology and Innovation Policy.

[46] Amini Z., Arasti Z., Bagheri A. (2018). Identifying social entrepreneurship competencies of managers in social entrepreneurship organization in healthcare sector. J. Global Entrep. Res..

[47] He A. (2018). Manoeuvring within a fragmented bureaucracy: Policy entrepreneurship in China’s local healthcare reform. China Q..

[48] Wilden R., Garbuio M., Angeli F., Mascia D. (2018). Entrepreneurship in Healthcare.

[49] Eriksson N., Ujvari S. (2015). Fiery Spirits in the context of institutional entrepreneurship in Swedish healthcare. J. Health Org. Manag..

[50] Salvino R., Tasto M., Randolph G. (2014). Entrepreneurship and the consequences of healthcare policy. J. Entrep. Pub. Policy.

[51] Ramadi K.B., Srinivasan S., Atun R. (2019). Health diplomacy through health entrepreneurship: Using hackathons to address Palestinian-Israeli health concerns. BMJ Glob. Health.

[52] Orton S., Umble K., Zelt S., Porter J., Johnson J. (2007). Management Academy for Public Health: Creating Entrepreneurial Managers. Am. J. Public Health.

[53] Becker E.R.B., Chahine T., Shegog R. (2019). Public health entrepreneurship: A novel path for training future public health professionals. Front. Public Health.

[54] Hatef E., Sharfstein J.M., Labrique A.B. (2018). Innovation and entrepreneurship: Harnessing the public health skill set in a new era of health reforms and investment. J. Public Health Manag. Pract..

[55] Duncan W.J., Ginter P.M., Rucks A.C., Jacobs T.D. (1988). Intrapreneurship and the reinvention of the corporation. Bus. Horiz..

[56] Cullen M.D.M., Calitz A.P., Allen K.I. Assessing intrapreneurship in a pharmaceutical manufacturing organisation in South Africa. Proceedings of the International Business Conference 2018 (IBC).

[57] Covin J.G., Miller D. (2014). International entrepreneurial orientation: Conceptual considerations, research themes, measurement issues, and future research directions. Entrep. Theory Pract..

[58] Kuratko D.F., Hornsby J.S., Covin J.G. (2014). Diagnosing a firm’s internal environement for corporate entrepreneurship. Bus. Horiz..

[59] Heinonen J., Korvela K. How about measuring intrapreneurship?. Proceedings of the 33rd EISB Conference.

[60] Weiss K.B., Sullivan S.D. (2001). The health economics of asthma and rhinitis. I assessing the economics impact. J. Allergy Clin. Immun..

[61] Ostrom E. (2005). Unlocking Public Entrepreneurship and Public Economies.

[62] Lages M., Marques C.S., Ferreira J.J., Ferreira F.A. (2017). Intrapreneurship and firm entrepreneurial orientation: Insights from the health care service industry. Intern. Entrep. Manag. J..

[63] Zahra S., Hayton J. (2008). The effect of international venturing on firm performance: The moderating influence of absorptive capacity. J. Bus. Vent..

[64] Huse M. (2007). Boards, Governance and Value Creation: The Human Side of Corporate Governance.

[65] Teng B. (2007). Corporate entrepreneurship activities through strategic alliances: A resource-based approach toward competitive advantage. J. Manag. Stud..

[66] Antončič B., Hisrich R.D. (2003). Clarifying the intrapreneurship concept. J. Small Bus. Enterp. Develop..

[67] Gupta M.R., Barman T.R. (2010). Health, infrastructure, environment and endogenous growth. J. Macroeconom..

[68] Chen C. (2018). Measuring the efficiency of regional entrepreneurship systems—An application of dynamic network DEA on Taiwan’s counties and cities. Croat. Operat. Res. Rev..

[69] Stuetzer M., Audretsch D.B., Obschonka M., Gosling S.D., Rentfrow P.J., Potter J. (2018). Entrepreneurship culture, knowledge spillovers and the growth of regions. Reg. Stud..

[70] Stuetzer M., Obschonka M., Audretsch D.B., Wyrwich M., Rentfrow P.J., Coombes M., Shaw-Taylor L., Satchell M. (2016). Industry structure, entrepreneurship, and culture: An empirical analysis using historical coalfields. Euro. Econ. Rev..

[71] Obschonka M., Schmitt-Rodermund E., Silbereisen R.K., Gosling S.D., Potter J. (2013). The regional distribution and correlates of an entrepreneurship-prone personality profile in the United States, Germany and the United Kingdom: A sociological perspective. J. Personal. Soc. Pshych..

[72] Obschonka M., Stuetzer M., Gosling S.D., Rentfrow P.J., Lamb M.E., Potter J., Audretsch D.B. (2015). Entrepreneurial regions: Do macro-psychological cultural characteristics of regions help solve the ‘knowledge paradox’ of economics?. PLoS ONE.

[73] Acemoglu D., Johnson S., Robinson J.A. (2001). The colonian origins of comparative development: An empirical investigation. Am. Econ. Rev..

[74] North D. Transaction Costs Through Time. https://econpapers.repec.org/paper/wpawuwpeh/9411006.htm.

[75] Baumol W. (1990). Entrepreneurship: Productive, unproductive, and destructive. J. Political Econ..

[76] Audretsch D.B., Kuratko D.F., Link A.N. (2015). Making sense of the elusive paradigm of entrepreneurship. Small Bus. Econ..

[77] Ireland R.D., Covin J.G., Kuratko D.F. (2009). Conceptualizing corporate entrepreneurship strategy. Entrep. Theory Pract..

[78] Shane S., Venkataraman S. (2000). The promise of entrepreneurship as a field of research. Acad. Manag. Rev..

[79] Sharir M., Lerner M. (2006). Gauging the success of social ventures initiated by individual social entrepreneurs. J. World Bus..

[80] Cromie S. (1987). Motivations of aspiring male and female entrepreneurs. J. Org. Behav..

[81] Markman G.D., Gianiodis P.T., Phan P.H., Balkin D.B. (2005). Innovation speed: Transferring university technology to market. Res. Policy.

[82] Bygrave W. (1989). The entrepreneurship paradigm I: A philosophical look at its research methodologies. Entrep. Theory Pract..

[83] Borkowski N., Kulzick R. (2006). Perspectives from the field: Will recent public policies reduce entrepreneurship in the healthcare industry. Intern. J. Public Adm..

[84] Ács Z., Gifford S. (1996). Innovation of entepreneurial firms. Small Bus. Econ..

[85] McKelvie A., Wiklund J. (2010). Advancing firm growth research: A focus on growth mode instead of growth rate. Entrep. Theory Pract..

[86] Lucas D.S., Fuller C.S. (2017). Entrepreneurship: Productive, unproductive, and destructive—Relative to what?. J. Bus. Vent. Insights.

[87] Lumpkin G.T., Dess G.G. (1996). Clarifying the entrepreneurial orientation construct and linking it to performance. Acad. Manag. Rev..

[88] Covin J.G., Slevin D.P. (1991). A conceptual model of entrepreneurship as firm behavior. Entrep. Theory Pract..

[89] Miller D. (1987). Strategy making and structure: Analysis and implications for performance. Acad. Manag. J..

[90] Burgelman R.A., Maidique M.A., Wheelwright S.C. (1996). Strategic Management of Technology and Innovation.

[91] Piron C. (2017). Key conditions for successful serial entrepreneurship in healthcare. Healthc. Pap..

[92] Kuratko D.F., Montagno R.V., Hornsby J.S. (1990). Developing an intrapreneurial assessment instrument for an effective corporate entrepreneurial environemnt. Strat. Manag. J..

[93] Rauch A., Wiklund J., Lumpkin G.T., Frese M. (2009). Entrepreneurial orientation and business performance: An assessment of past research and suggestions for the future. Entrep. Theory Pract..

[94] Slevin P., Terjesen A. (2011). Entrepreneurial orientation: Reviewing three papers and implications for further theoretical and methodological development. Entrep. Theory Pract..

[95] Tang Z., Kreiser P., Marino L., Dickson P., Weaver K. (2008). A hierarchical perspective of the dimensions of entrepreneurial orientation. Intern. Entrep. Manag. J..

[96] Van Praag C.M., Versloot P.H. (2007). What is the value of entrepreneurship? A review of recent research. Small Bus. Econ..

[97] Guth W., Ginsberg A. (1990). Corporate entrepreneurship. Strat. Manag. J..

[98] Miller D. (1983). The correlates of entrepreneurship in three types of firms. Manag. Sci..

[99] Hughes M., Morgan R.E. (2007). Deconstructing the relationship between entrepreneurial orientation and business performance at the embryonic stage of firm growth. Ind. Mark. Manag..

[100] Holt D.H., Englewood Cliffs N.J. (1992). Entrepreneurship: New Venture Creation.

[101] Hisrich R.D., Peters M.P. (1984). Internal venturing in large corporations: Frontiers of Entrepreneurship Research.

[102] MacMillan I.C., Block Z., Narasimha P.S. (1986). Corporate venturing: Alternatives, obstacles encountered, and experience effects. J. Bus. Vent..

[103] Schollhammer H. (1981). The efficacy of internal corporaye entrepreneurship strategies. Front. Entrep. Res..

[104] Chua J.H., Chrisman J.J., Sharma P. (1999). Defining the family business by behavior. Entrep. Theory Pract..

[105] Stopford J.M., Baden-Fuller C.W. (1994). Creating corporate entrepreneurship. Strat. Manag. J..

[106] Zahra S. (1991). Predictors and financial outcomes of corporate entrepreneurship: An exploratory study. J. Bus. Vent..

[107] Ireland R.D., Kuratko D.F., Morris M.H. (2006). A health audit for corporate entrepreneurship: Innovation at all levels, Part I. J. Bus. Strat..

[108] Antončič A.A., Antončič B. (2011). Employee satisfaction, intrapreneurship and firm growth: A model. Ind. Manag. Data Syst..

[109] Sakellarides C. (2008). A Lisbon agenda on health innovation. Euro. J. Public Health.

[110] Lehoux P., Pacifico Silva H., Pozelli Sabio R., Roncarolo F. (2018). The unexplored contribution of responsible innovation in health to sustainable development goals. Sustainability.

[111] Silva H.P., Lehoux P., Miller F.A., Denis J.L. (2018). Introducing responsible innovation in health: A policy-oriented framework. Health Res. Policy Syst..

[112] Di Fabio A., Bucci O., Gori A. (2016). High entrepreneurship, leadership and professionalism (HELP): Toward and integrated, empirically based perspective. Front. Psychol..

[113] Zahra S.A., Garvis D.M. (2000). International corporate entrepreneurship and firm performance: The moderating effect of internatioanl environmental hostility. J. Bus. Vent..

[114] Lumpkin G.T., Dess G.G. (1997). Proactiveness versus competitive aggressiveness: Testing apart key dimensions of an entrepreneurial orientation. Front. Entrep. Res..

[115] Morris M. (2015). Entrepreneurial intensity. Wiley Encyclopedia of Management.

[116] Brockhaus R. (1980). Risk taking propensity of entrepreneurs. Acad. Manag. J..

[117] Mintzberg H. (1973). Strategy-making in three modes. Calif. Manag. Rev..

[118] Miles R.E., Snow C.C., Meyer A.D., Coleman H.J. (1978). Organizational strategy, structure, and process. Acad. Manag. Rev..

[119] Kreiser P.M., Marino L.D., Dickson P., Weaver K.M. (2010). Cultural influences on entrepreneurial orientation: The impact of national culture on risk taking and proactiveness in SMEs. Entrep. Theory Pract..

[120] Dutta U. (2019). Indigenous Health Organizing at the Margins: Creating Access to Health by Building Health Infrastructure. Health Commun..

[121] Ostrom E. (1965). Public Entrepreneurship: A Case Study in Ground Water Basin Management. Ph.D. Dissertation.

[122] Pinchott G. (1985). Intrapreneuring: Why You Don’t Have to Leave the Corporation to Become an Entrepreneur.

[123] Knight G.A. (1997). Cross-cultural reliability and validity of a scale to measure firm entrepreneurial orientation. J. Bus. Vent..

[124] Kusa R. (2016). Measuring entrepreneurial orientation in the social context. Entrep. Bus. Econ. Rev..

[125] Petković S., Sorak S. (2019). Effects of the establishment of entrepreneurial orientation on the performances of small and medium enterprises in transition countries: Empirical evidences from Bosnia and Herzegovina. Zagreb Intern. Rev. Econ. Bus..

[126] Edmond V.P., Wiklund J. (2010). The historical roots of entrepreneurial orientation research. Historical Foundations of Entrepreneurship Research.

[127] George B.A., Marino L. (2011). The epistemology of entrepreneurial orientation: Conceptual formation, modeling, and operationalization. Entrep. Theory Pract..

[128] Arbaugh B.J., Asarta C.J., Hwang A., Fornaciari C.J., Charlier S.D. (2019). Business and Management Education Research: Developing and Assessing Research Streams Using Legitimation Code Theory. Acad. Manag. Learn. Educ..

[129] Kreiser P.M., Marino L.D., Weaver K.M. (2002). Assessing the psychometric properties of the entrepreneurial orientation scale: A multi-country analysis. Entrep. Theory Pract..

[130] Yoo S.-J., Bygrave W.D., Brush C.G., Davidsson P., Green G.P., Reynolds P.D., Sapienca H.J. (2001). Entrepreneurial orientation, environment scanning intensity, and firm performance in technology-based SMEs. Frontiers of Entrepreneurship Research.

[131] Brown T.E., Davidsson P., Wiklund J. (2001). An operationalization of Stevenson’s conceptualization of entrepreneurship as opportunity-based firm behavior. Strat. Manag. J..

[132] Lee S.M., Peterson S.J. (2000). Culture, Entrepreneurial Orientation, and Global Competitiveness. J. World Bus..

[133] Wiklund J. (1999). The sustainability of the entrepreneurial orientation—Performance relationship. Entrep. Theory Pract..

[134] Becherer R.C., Maurer J.G. (1999). The proactive personality disposition and entrepreneurial behavior among small company presidents. J. Small Bus. Manag..

[135] Lee C., Lee K., Pennings J.M. (2001). Internal capabilities, external networks, and performance: A study of technology bases ventures. Strat. Manag. J..

[136] Miller D., Toulouse J.M. (1986). Chief executive personality and corporate strategy and structure in small firms. Manag. Sci..

[137] Smart D.T., Conant J.S. (1994). Entrepreneurial orientation, distinctive marketing competencies and organizational performance. J. Appl. Bus. Res..

[138] Haq Z., Hafeez A., Zafar S., Ghaffar A. (2017). Dynamics of evidence-informed health policy making in Pakistan. Health Policy Plan..

[139] Beaulieu M., Lehoux P. (2018). Emerging health technology firms’ strategies and their impact on economic and healthcare system actors: A qualitative study. J. Innov. Entrep..

[140] Gartner W. (1990). What are we talking about when we talk about entrepreneurship?. J. Bus. Vent..

[141] Dempster G., Kluver J. (2019). Institutional entrepreneurship in health management: A survey experiment on appreciative inquiry. Stud. Bus. Econ..

[142] Robu R.G. (2019). Challenges in measuring entrepreneurship and entrepreneurial environment with an impact on regional economic development: A theoretical review. Annals of the “Constantin Brâncuşi” University of Târgu Jiu. Econ. Ser..

[143] Barzilay E.J., Vandi H., Binder S., Udo I., Ospina M.L., Ihekweazu C., Bratton S. (2018). Use of the staged development tool for assessing, planning, and measuring progress in the development of national public health institutes. Health Secur..

[144] Lafuente E., Szerb L., Ács Z.J. (2016). Country level efficiency and national systems of entrepreneurship: A data envelopment analysis approach. J. Techn. Trans..

[145] Di Fabio A. (2014). Intrapreneurial self-capital: A new construct for the 21st century. J. Employ. Couns..

[146] Luthans F., Youssef C.M. (2007). Emerging positive organizational behavior. J. Manag..

[147] Bateman T.S., Crant J.M. (1993). The proactive component of organizational behavior: A measure and correlates. J. Org. Behav..

[148] Rosenberg M. (2015). Society and the Adolescent Self-Image.

[149] Diener E.D., Emmons R.A., Larsen R.J., Griffin S. (1985). The satisfaction with life scale. J. Personal. Assess..

[150] Lloyd-Williams F., Bromley H., Orton L., Hawkes C., Taylor-Robinson D., O’Flaherty M., Rayner M. (2014). Smorgasbord or symphony? Assessing public health nutrition policies across 30 European countries using a novel framework. BMC Public Health.

[151] Ahmad N., Hoffman A. (2008). A Framework for Addressing and Measuring Entrepreneurship.

[152] Grujić V. (1998). Metode Mjerenja Zdravlja i Zdravstvenog Stanja Stanovnitšva.

[153] Martinov-Cvejin M. (1998). Mjerenje Efikasnosti i Kvaliteta Rada Zdravstvenih Ustanova.

[154] Milosavljević N. (1993). Uvod. Definicija Ekonomike Zdravstva. Ekonomika Zdravstva kao Instrument Zdravstvene Politike.

[155] Pavić Ž. (2010). Zdravstvo i Zdravstveni System.

[156] Leszczensky L., Wolbring T. (2018). How to deal with reverse causality using panel data? Recommendations for researchers based on a simulation study. Soc. Methods Res..

[157] Vaisey S., Miles A. (2017). What you can—And can’t—Do with three-wave panel data. Soc. Methods Res..

[158] Loi M., Rodrigues M. (2012). A Note on the Impact Evaluation of Public Policies: The Counterfactual Analysis.

[159] Ghani E., Kerr W.R., O’Connell S. (2014). Spatial determinants of entrepreneurship in India. Reg. Stud..

[160] Fritsch M., Falck O. (2007). New business formation by industry over space and time: A multidimenstional analysis. Reg. Stud..

[161] Card D. (1999). The causal effect of education on earnings. Handb. Labor Econ..

[162] Hausman J.A. (1978). Specification tests in econometrics. Econom. J. Econom. Soc..

[163] Wooldridge J.M. (2010). Econometric Analysis of Cross Section and Panel Data.

[164] Yoo K. (2015). A comparative study of cultural dimension as an influencing factor to entrepreneurial orientation. Interat. J. Bus. Soc. Sci..

[165] Lumpkin G.T., Cogliser C.C., Schneider D.R. (2009). Understanding and measuring autonomy: An entrepreneurial orientation perspective. Entrep. Theory Pract..

[166] Krauss S.I., Frese M., Friedrich C., Unger J.M. (2005). Entrepreneurial orientation: A psychological model of success among southern African small business owners. Eur. J. Work. Organ. Psychol..

[167] Stevenson H.H., Jarillo J.C. (1990). A paradigm of entrepreneurship: Entrepreneurial management. Strat. Manag. J..

[168] Lin H.C. (2006). Building competitive advantage: The roles of upper echelons and competitive aggressiveness. Acad. Manag. Proc..

[169] Szerb L., Lafuente E., Horváth K., Páger B. (2019). The relevance of quantity and quality entrepreneurship for regional performance: The moderating role of the entrepreneurial ecosystem. Reg. Stud..

[170] Petković S., Jaeger C., Sašić B. (2016). Challenges of small and medium sized companies at early stage of development: Insights from Bosnia and Herzegovina. Manage.

[171] Müller S. (2016). A Progress Review of Entrepreneurship and Regional Development: What Are the Remaining Gaps?. Eur. Plan. Stud..

[172] Richardson A., Audretsch D.B., Aldrige T., Audretsch D., Link A. (2016). Motivating entrepreneurship and innovative activity: Analyzing US policies and programs. Essay in Public Sector Entrepreneurship.

[173] Acemoglu D., Johnson S., Robinson J.A. (2002). Reversal of fortune: Geography and institutions in the making of the modern world income distribution. Q. J. Econ..

[174] Baumol W., Audretsch D., Storm R. (2009). Entrepreneurship, trade competition and the explosion of world trade. Entrepreneurship and Openess: Theory and Evidence.

[175] De Soto H. (2000). The Mystery of Capital: Why Capitalism Triumphs in the West and Fails Everywhere Else.

[176] Kuratko D.F., Audretsch D.B. (2013). Clarifying the domains of corporate entrepreneurship. Intern. Entrep. Manag. J..

[177] Hornsby J.S., Kuratko D.F., Zahra S.A. (2002). Middle managers’ perceptions of the internal environment for corporate entrepreneurship: Assessing a measurement scale. J. Bus. Vent..

[178] Kuratko D.F., Ireland R.D., Hornsby J.S. (2001). Improving firms performance through entrepreneurial actions: Acordia’s corporate entrepreneurship strategy. Acad. Manag. Perspect..

[179] Zahra S.A., Covin J.G. (1995). Contextual influences on the corporate entrepreneurship-performance relationship: A longitudinal analysis. J. Bus. Vent..

[180] North D. (1990). Institutions, Institutional Change and Economic Performance.

[181] North D.C., Thomas R.P. (1973). The Rise of the Western World: A new Economic History.

[182] Guo K.L. (2003). Applying Entrepreneurship to Health Care Organizations. N. Engl. J. Entrep..

[183] Smith R., Guiness L., Wiseman V. (2011). Macroeconomics, globalization and health. Understanding Public Health—Introduction to Health Economics.

